# Genetic database software as medical devices

**DOI:** 10.1002/humu.23621

**Published:** 2018-10-11

**Authors:** Adrian Thorogood, Seydina B. Touré, Johan Ordish, Alison Hall, Bartha Knoppers

**Affiliations:** ^1^ Centre of Genomics and Policy McGill University Montreal Canada; ^2^ PHG Foundation University of Cambridge Cambridge UK

**Keywords:** data sharing, European Union, FDA, genomics, law, medical device, public genetic variant databases, regulation, software

## Abstract

This article provides a primer on medical device regulations in the United States, Europe, and Canada. Software tools are being developed and shared globally to enhance the accessibility and usefulness of genomic databases. Interactive software tools, such as email or mobile alert systems providing variant classification updates, are opportunities to democratize access to genomic data beyond laboratories and clinicians. Uncertainty over the reliability of outputs, however, raises concerns about potential harms to patients, especially where software is accessible to lay users. Developers may also need to contend with unfamiliar medical device regulations. The application of regulatory controls to genomic software could improve patient and user safety, but could also stifle innovation. Legal uncertainty for developers is compounded where software applications are made available globally (implicating multiple regulatory frameworks), and directly to lay users. Moreover, there is considerable uncertainty over the application of (evolving) medical device regulations in the context of both software and genetics. In this article, criteria and examples are provided to inform determinations of software as medical devices, as well as risk classification. We conclude with strategies for using genomic communication and interpretation software to maximize the availability and usefulness of genetic information, while mitigating the risk of harm to users.

## Abstract 2

This article provides a primer on medical device regulation of software that interprets and exchanges genomic data. We compare tests for determining if software qualifies as a medical device across the United States, Europe, and Canada, as well as risk classifications and regulatory controls. Medical device regulation of both software and genetic tools remains uncertain, raising competing concerns: insufficient regulation allows low quality outputs to undermine patient care, while excessive regulation stifles development of innovative tools delivering precision medicine.

## 1 INTRODUCTION

Physicians, clinical laboratories, and public human genetic variant databases (hereafter “variant databases”) all contribute to an interpretive “supply chain” supporting diagnosis and personalized care. Variant databases provide access to collective but rapidly evolving knowledge about associations between genomes and disease. Databases are increasingly used to inform clinical decision making and regulatory approvals of tests. In turn, policy makers are exploring ways to regulate the quality of these databases (U.S. Food and Drug Administration, 2018d). At the same time, more variant databases are also integrating innovative software tools to improve accessibility, usefulness, and interactivity. Such tools span from data retrieval, query, notification, and visualization functions, to risk prediction enabled by artificial intelligence. The United States National Center for Biotechnology Information's ClinVar database, for example, is developing processes to provide email alerts when a variant assertion changes (an assertion is an “informed assessment of a genotype–phenotype correlation [or lack thereof],” such as benign, pathogenic, or drug resistant). The BRCAExchange.org database, which pools publicly available knowledge on correlations between BRCA1/2 gene variants and breast and ovarian cancer, has developed a mobile app that provides a notification to patients interested in “following” their variant, and knowing if assertions change over time. In the era of big data, healthcare decision makers are faced with an unprecedented deluge of molecular and health‐related data. Software will no doubt play a central role in the future of personalized medicine. Genomic communication software tools may also help to democratize access and make genetic data more useful and intelligible to patients and the lay public. Other tools enable risk prediction or stratification: the Breast and Ovarian Analysis of Disease Incidence and Carrier Estimation Algorithm (Cambridge University), for example, calculates the individual risk level for developing breast or ovarian cancer, based on their family history and other data. These tools join a growing ecosystem of publicly available software (i.e., distributed freely by database or third‐party developers on the web or app stores) that supports the sharing and interpretation of genetic data.

Publicly available genomic software tools have the potential to improve the quality, intelligibility, and currency of data available to inform health‐related decisions by health professionals and lay users. Because of the risk that inaccurate outputs could harm patients, users need assurances that software is of high quality. The global “distribution” of software to clinicians and patients over the internet also raises a pressing legal question for developers: when does a genomic software application qualify as a regulated medical device? This paper provides an international primer focused exclusively on medical device (MD) regulation for genomic communication and interpretation software. In doing so, it draws from the harmonizing guidance of the International Medical Device Regulators Forum (IMDRF), as well as the globally important US Food and Drug Administration's medical device regulations, the new European Union Medical Device Regulation (EU MDR) and European Union In Vitro Diagnostic Device Regulation (EU IVDR), and the Canadian Medical Devices Regulations. We discuss these regulatory frameworks generally, while highlighting salient differences. First, we define the regulatory schemes for medical and in vitro diagnostic devices. Second, we provide criteria, exceptions, and examples to guide the determination of when publicly available software is a MD. Third, we discuss factors that determine the risk classification of MDs, and how higher classification ties to more burdensome general, pre‐ and postmarket requirements. To conclude, we recommend strategies for ensuring and demonstrating that publicly available precision medicine software applications are safe and effective when used to support the predictive testing, diagnosis, and treatment of patients. While we focus on software developed to enhance the interactivity of variant databases in support of precision medicine, our discussion will also be relevant to other genomic software tools, including clinical decision support intended to provide users “with knowledge and person‐specific information, intelligently filtered or presented at appropriate times, to enhance health and health care” (Senger & O'Leary, 2018), risk prediction algorithms (Hall et al., 2018), and in silico prediction tools (Prawira, Pugh, Stockley, & Siu, 2017). Our discussion is limited to the quality and regulation of genomic *software*. We do not address fundamental policy, technical, and possibly legal questions related to the quality of the underlying *data* shared by variant databases, as neither information alone nor the exercise of clinical judgment is subject to medical or in vitro diagnostic device regulation.

## 2 MEDICAL DEVICES, IN VITRO DIAGNOSTIC DEVICES, AND SOFTWARE

From privacy, to security, to consumer protection, many regulatory schemes potentially apply to genetic database software, and it may be complex for developers and users to navigate those that apply. In the United States, next‐generation sequencing technologies are for the most part regulated under Clinical Laboratory Improvement Amendments as laboratory‐developed tests (LDTs), though a number of cancer panels have recently been approved under MD regulations (U.S. Food and Drug Administration, 2017e). Both Congress and the Food and Drug Administration (FDA) have indicated that the FDA may yet become more involved in regulation of LDTs (Gibbs, 2018). In the EU, genomic data that can identify an individual is within the scope of and regulated by the General Data Protection Regulation. While each of these schemes presents its own set of policy challenges, we focus our discussion on MD and in vitro diagnostic device regulations. Under these schemes, software may be regulated when it falls under the relevant statutory definition in each jurisdiction (See Table 1).

**Table 1:** Definitions of medical device and in vitro diagnostic device

Medical device		
United States	Federal Food, Drug, and Cosmetic Act (FD&C) Section 201(h)	“An instrument […] which is […] intended for use in the diagnosis of disease or other conditions, or in the cure, mitigation, treatment, or prevention of disease […] which does not achieve its primary intended purposes through chemical action within or on the body of man or other animals and which is not dependent upon being metabolized for the achievement of its primary intended purposes. The term “device” does not include software functions excluded pursuant to section 520(o).”
European Union	Regulation (EU) 2017/745 (MDR) Article 2(1)	‘Medical device’ means any instrument […] software, […] or other article intended by the manufacturer to be used, alone or in combination […] for one or more of the following specific medical purposes: ‐ diagnosis, prevention, monitoring, prediction, prognosis, treatment or alleviation of disease, ‐ […] ‐ providing information by means of in vitro examination of specimens derived from the human body […], and which does not achieve its principal intended action by pharmacological, immunological or metabolic means, in or on the human body, but which may be assisted in its function by such means.
Canada	Food and Drugs Act Section 2	Device means an instrument, […] or other similar article, or an in vitro reagent[…] that is manufactured, sold or represented for use in (a) diagnosing, treating, mitigating or preventing a disease, disorder or abnormal physical state, or any of their symptoms […], […] however, it does not include such an instrument […] that does any of the actions referred to in paragraphs (a) to (e) solely by pharmacological, immunological or metabolic means or solely by chemical means in or on the body […].
IMDRF	Software as a Medical Device (SaMD): Key Definitions Section 5.2.1	‘Medical device’ means any instrument, […], software, […] other similar or related article, intended by the manufacturer to be used, alone or in combination […] for one or more of the specific medical purpose(s) of: □ diagnosis, prevention, monitoring, treatment or alleviation of disease, […] □ providing information by means of in vitro examination of specimens derived from the human body; and does not achieve its primary intended action by pharmacological, immunological or metabolic means […].

In vitro diagnostic device		
United States	21 CFR 809.3 (a)	“*In vitro diagnostic products* are those reagents, instruments, and systems intended for use in the diagnosis of disease or other conditions, including a determination of the state of health, in order to cure, mitigate, treat, or prevent disease or its sequelae. Such products are intended for use in the collection, preparation, and examination of specimens taken from the human body […].”
European Union	Regulation (EU) 2017/746 (IVDR) Article 2(2)	‘in vitro diagnostic medical device’ means any medical device which is a reagent, […], instrument, […], software or system, whether used alone or in combination, intended by the manufacturer to be used in vitro for the examination of specimens […], solely or principally for the purpose of providing information on one or more of the following: (a) concerning a physiological or pathological process or state; (b) concerning congenital physical or mental impairments; (c) concerning the predisposition to a medical condition or a disease; […]
Canada	Medical Devices Regulations Section 1	In vitro diagnostic device or IVDD means a medical device that is intended to be used in vitro for the examination of specimens taken from the body.
IMDRF	Definition of the Terms ‘Medical Device’ and ‘In Vitro Diagnostic (IVD) Medical Device’ Section 5.2	‘In Vitro Diagnostic (IVD) medical device’ means a medical device, whether used alone or in combination, intended by the manufacturer for the in‐vitro examination of specimens derived from the human body solely or principally to provide information for diagnostic, monitoring or compatibility purposes.

The primary qualifying criterion of a MD or an in vitro diagnostic MD (IVD) is a manufacturer's “intended use” (discussed below). The basic premise of MD/IVD regulation is “that the devices should work as intended … a manufacturer must be able to provide a reasonable assurance of a device's safety and effectiveness under the intended conditions of use” (Senger & O'Leary, 2018). In the United States, regulation of MDs and IVDs remain procedurally similar. Indeed, in its overview of IVD regulation, the FDA indicates that “IVDs are medical devices [and] like other medical devices, […] are subject to premarket and postmarket controls” (U.S. Food and Drug Administration, 2018c). In addition, both are generally subject to regulatory control that is proportional to risk class. In the EU, MDs and IVDs are subject to similar requirements—the *MDR* and *IVDR* being separate regulations but sharing many articles and annexes. The apparent rationale for establishing distinct regulations for IVDs is based on regulatory disparities with other MDs in terms of “risk classification, conformity assessment procedures, and clinical evidence” (EU IVDR Recital 6).

Software used alone, or in combination with a MD, can qualify as a regulated MD in all three jurisdictions if intended to be used for one or more specific medical purposes (IMDRF SaMD Working Group, 2013). Software can be defined as a set of instructions or algorithms that processes input data and creates output data (MEDDEV 2.1/6—2016). Regulated software may be integral to a MD, used in the manufacture or maintenance of a MD, or stand alone. Previous guidance relating to the EU MDs Directive uses the terms accessories, modules, and stand‐alone MDs (European Commission, 2016). Regulations are platform agnostic. As such, mobile and cloud‐based apps may qualify as regulated MD/IVDs. Traditionally, regulators have been most concerned with software embedded in hardware MDs that could physically harm individuals, such as the Therac‐25 radiation incident, where defective software had caused several fatal X‐ray overdoses (Leveson & Turner, 1993). However, their perspective has been broadened by harms caused by other software. These would include stand‐alone software, which is distant from the patient. Risk assessment is complicated, however, by a number of factors, such as: the ease of availability across platforms; the complex interconnection of systems and datasets; and challenges related to software development, updates, and distribution (IMDRF Software as a Medical Device (SaMD) Working Group, 2014). Consequently, in the EU context at least, it is important to determine whether a specific device counts as an MD or IVD, as this determination has downstream effects for how a device is classified and what information is required to evidence safety and efficacy.

For genetic variant database software, this determination is especially important, as it is unclear whether they might qualify as MDs or IVDs. If regarded as a natural extension of sequencing machines, they would certainly count as IVD devices. The MEDDEV 2.1/6 interpretation, for example, considers software to be an IVD if “intended to capture and analyze together results obtained for one patient by one or more IVD […] to provide information falling within the definition of an IVD MD, for example, differential diagnosis.” This guidance goes on to provide examples of IVD devices, one of which being “software that integrates genotype of multiple genes to predict risk of developing a disease or medical condition.” Yet, the US, the EU, and Canada all recognize the examination of “specimens” as the core element to the definition of IVDs (Table 1). It is our contention that to count the data extracted from sequenced data as a “specimen” itself is a stretch in interpretation. Doing so would dramatically expand the scope of what counts as an IVD device, given the fact that several devices operate on data derived from the examination of specimens at some stage. To retain a sensible distinction between MDs and IVDs, it makes sense to interpret “specimen” narrowly, excluding genetic variant database software from the definition of IVDs if they are separate to sequencers.

The application of regulations to software reflects a fundamental policy tension. On one hand, variant databases and developers who make software tools publicly available could potentially improve the quality, availability, currency, and usefulness of genomic knowledge informing diagnosis and treatment. On the other hand, inaccurate or outdated software outputs may lead to harmful healthcare decisions by clinicians or lay users, and raise concerns about database and developer liability. Under‐regulation might expose the public to undue risk. Conversely, onerous regulatory controls, or simply uncertainty about what rules apply, can discourage the development and distribution of innovative software. Unsurprisingly, this policy uncertainty translates into legal uncertainty as to whether a given software will be regulated, compounded where software is distributed globally, engaging numerous regulatory regimes (that are also shifting unpredictably in an attempt to keep pace with rapid technological development).

## 3 MANUFACTURER'S “INTENDED USE”

At the highest level of generality, an instrument or software is an MD or IVD if its intended use is medical in nature (e.g., diagnosis, treatment, or prevention of disease) (Table 1). Most statutory definitions of MDs make explicit reference to the intended use of the product as a predefining characteristic. Specifically, the FDA defines “intended use” according to the manufacturer's “objective intent.” This definition flows from s. 801.4 of the US Code of Federal Regulations, which provides that “intended uses” are determined by expressions (any labeling, advertising, or statements), or by the circumstances of the device's distribution or use (known to the manufacturer) (U.S. Code of Federal Regulations, 2017). Still, the reader should bear in mind that, at the time of writing, there is ongoing controversy surrounding the FDA's application of this objective intent standard for determining a manufacturer's labeling requirements. In short, the FDA considered expanding s. 801.4 to allow investigating the “totality of evidence” to establish that a manufacturer objectively intended for a device to be used for a purpose other than that for which it had been approved, to then require the said manufacturer to update its labeling (U.S. Food and Drug Administration, 2017b). This interpretation appears to go beyond the currently applicable standard, which focuses solely on a manufacturer's knowledge of unapproved use, leaving industry members confused as to the role of knowledge alone in the determination of intended use (Kahan et al., 2017). In response, the FDA suspended these amendments. This controversy demonstrates the potential tension between manufacturers who require clarity about the obligations being placed upon them and regulators who wish to prevent regulation being evaded simply by avoiding overt medical claims.

Similarly, under the EU regulations, a device must have an intended medical purpose to qualify as a MD or IVD (though MDs listed in Annex IX of the EU MDR automatically qualify). The EU IVDR has no such equivalent list. Again, under art. 2 of the MD and IVD regulations, it is the manufacturer's intention that is determinative. Pursuant to arts. 2(12) and 2(11) of the EU MDR and EU IVDR, respectively, intent has traditionally been determined primarily by the labeling, advertising material, instructions, and other such materials to construe intended purpose. The Court of Justice of the European Union (CJEU) has elaborated on “intended use” in the context of the Medical Device Directive (93/42). In *C‐219/11, Brain Products GmbH v BioSemi VOF EU:C:2012:742*, the court delivered a preliminary ruling on whether a device that enabled human brain activity to be recorded qualified as a MD (C‐219/11, 2012). The CJEU highlighted that if software is to qualify as a MD in its own right; its manufacturer must specifically intend that the device be used for one (or more) medical purpose set out in the definition of MD. In other words, it is not enough that software be used for general purposes in a healthcare setting. The recent decision in *C‐329/16, Syndicat national de l'industrie des technologies médicales (Snitem) v Premier minister, Ministre des Affaires sociales et de la Santé EU:C:2017:947* further clarified the concept of intended use (C‑329/16, 2017). The preliminary ruling in *Snitem* concerned prescription assistance software and whether this software qualified as a MD. While the court's reasoning largely followed *Brain Products*, the court relied heavily on the function of the device rather than any advertising or instructional material, saying:

“I[t] follows that software, of which at least one of the functions makes it possible to use patient‐specific data for the purposes, inter alia, of detecting contraindications, drug interactions and excessive doses, is, in respect of that function, a medical device…”

In all likelihood, the *Snitem* judgment suggests that the court will increasingly emphasize the function of the device to determine the intended use of the device.

Compared to the United States and Europe, the role of “intended use” is less direct in Canada. Of all the jurisdictions studied in this article, Canada is the only one without explicit reference to “intended use” in the regulatory definition of a MD (though the definition of IVD does refer to “intended use”). Regardless, the intended use of a MD is the primary criterion for determining the device's risk class (Health Canada, 2010, 2015). Although the practical effect of this discrepancy remains unclear to us, it highlights how the same regulatory standard or term may be used differently across jurisdictions. To illustrate, in the United States, a manufacturer might be required to amend the labeling of its device if the said device was used in a manner that was not approved (under s. 801.4 of the CFR, and subject to the “knowledge” standard discussed previously). By contrast, in Canada, the use of a device in a manner not intended by the manufacturer does not affect the classification of the device—and presumably, applicable labeling requirements and regulatory controls (Health Canada, 2015). While the obvious follow‐up question should be what “not intended by the manufacturer” means in Canadian practice, this comparison nevertheless illustrates the differential effects of third‐party use on the labeling (and potentially the classification) of a device across United States and Canadian jurisdictions, complicating the calculus for software developers.

## 4 IS MY DATABASE SOFTWARE AN MD/IVD? CRITERIA AND EXCEPTIONS

Variant databases are often established to inform genetic medicine, so the natural assumption is that any software application these databases distribute will be a MD. There are, however, a number of relevant exceptions.

### 4.1 Library functions

Software that carries out “library functions” does not generally attract regulation. FDA guidance has long suggested that software carrying out “library” functions, such as storage, retrieval, and dissemination of information—traditionally carried out through textbooks and journals—is not regulated (U.S. Food and Drug Administration, 1987). The position in the EU is likely to be similar, with the MEDDEV 2.1/6 guidance clarifying that software that performs actions limited to storage, archival, communication, or simple searches are not MD/IVDs. This library function exception would seem to cover many of the preliminary software applications that may be distributed by variant databases. Lending support to this conclusion is the recent guidance for “FDA recognized” variant databases, which recognizes the library nature of variant databases (U.S. Food and Drug Administration, 2018d). The guidance defines an assertion as “the informed assessment of a genotype–phenotype correlation (or lack thereof) given the current state of knowledge for a particular variant” and adds that in “order to be FDA recognized, a variant database should not include any recommendations regarding clinical treatment or diagnosis. However, an assertion that states that a variant is clinically significant as an actionable mutation may be found within an FDA‐recognized genetic variant database.” Perhaps unsurprisingly, genetic data‐sharing tool developers appear to challenge or reject the view that their tools perform “interpretation”; rather, they characterize their tools as providing a “bridge” to scientific literature or annotation databases (e.g., PubMed, ClinVar, and SNPedia) (Nelson & Fullerton, 2018). Accordingly, some authors suggest that “[i]f health information is presented with the intent to educate, rather than diagnose or treat [or prevent], it will not be regulated as a device—even if consumers use the information to self‐diagnose” (Spector‐Bagdady & Pike, 2014).

### 4.2 Expert systems

The library function exception relates to a broader exception for expert or knowledge‐based systems “intended to involve competent human intervention before any impact on human health occurs (e.g., where clinical judgment and experience can be used to check and interpret a system's output)” (U.S. Food and Drug Administration, 1987). In connection, the 21st Century Cures Act (Cures Act) added s. 520(o)(1)(E) to the Food, Drug, and Cosmetic Act to exclude certain clinical decision support software functions from the definition of device. Among the conditions necessary for a statutory exemption, a given software must be intended for the purpose of enabling the healthcare professional to independently review the basis of the clinical recommendations made by the software (Cures Act, s. 520(o)(1)(E)(iii)). Subsequently, the FDA issued a draft guidance in which it clarifies that “[i]n order for the software function to be [exempted], the intended user should be able to reach the same recommendation on his or her own without relying primarily on the software function” (U.S. Food and Drug Administration, 2017c). The advent of increasingly complex and inscrutable algorithms, however, is “eroding” the meaningfulness of this exception, where reviewers cannot meaningfully second‐guess results (Senger & O'Leary, 2018). Some observers question whether the Cures Act's standard for excluding expert systems from FDA oversight is a workable standard as applied to machine‐learning software. Can regulators readily determine when “software explains its recommendations in a way that physicians can understand and critique?” or when “the manufacturer intended for its software to do so?” (Evans & Ossorio, 2018) This in turn raises concerns that the software labeling may be gamed by software developers, for example, by requiring physician supervision, eroding patient safety and shifting liability risks to physicians (Evans & Ossorio, 2018).

### 4.3 Wellness devices

Software for general purposes or software intended for lifestyle and well‐being purposes, is typically not actively regulated in the reviewed jurisdictions, even when used in a healthcare setting (see e.g., EU MDR Recital 19). In the United States, for instance, if the software in question is “intended for individuals to log, record, track, evaluate, or make decisions or behavioral suggestions related to developing or maintaining […] health and wellness,” this software escapes FDA jurisdiction, as it would be among the newly‐created exceptions under s. 3060 of the Cures Act (U.S. Food and Drug Administration, 2017a). Interestingly, even prior to the Cures Act amendments, the FDA exercised enforcement discretion over these products (U.S. Food and Drug Administration, 2015). As such, the Cures Act effectively crystallizes preexisting FDA policy into binding law. Likewise, in the EU, “well‐being” or “lifestyle” software are not MD/IVDs (EU MDR Recital 19 and IVDR Recital 17).

Interestingly, the recent developments in the United States have harmonized FDA and EU statutory exemptions. In the past, a key difference between the United States and European regulations was that the United States was discretion based (i.e., the FDA retaining jurisdiction over the medical mobile app but choosing not to exercise it), whereas in Europe, the mobile app would simply not be considered as an MD/IVD and would presumably lie outside the ambit of the EU MDR/IVDR (Recitals 19 and 17, respectively). Outside the strict domain of MD or IVD regulation, more general health app regulation has been explored in the EU by the Report of the Working Group on mHealth Assessment Guidelines. While this group is yet to find consensus, there has been a more general proposal to unify health technology assessment in the form of a new regulation (European Commission, 2018).

### 4.4 Laboratory‐developed tests/Health institution exemptions

Developers should also be aware of the regulatory framework of Laboratory‐Developed Tests (LDT) or, more commonly known in the EU, the health institution exemptions (HIE). Broadly defined, an LDT is a type of IVD “that is designed, manufactured, and used within a single laboratory” (U.S. Food and Drug Administration, 2018b). Briefly, LDTs have traditionally escaped regulation, and did so via various regulatory mechanisms. In the United States, this exemption is discretionary and nonstatutory (U.S. Food and Drug Administration, 2018b). In the EU, it is statutory (see Art. 5 para 5 in both EU IVDR and EU MDR). In Canada, LDT regulation is much less well‐defined, and has even been considered a loophole (Clifford, Dhalla, Miller, & Rousseau, 2017). In practical terms, therefore, variant database software that are LDTs will be in a similar situation as nondevice databases. Still, while ascertaining whether their device qualifies for the LDT/HIE exemption, public variant database software developers should remain mindful of the following observations. First, most publicly available software will not qualify as LDTs on account of their “public” availability. Second, the FDA is increasingly tightening the scope of the LDT exemption in order to exercise greater oversight (U.S. Food and Drug Administration, 2017b, 2017d). This is due in part to the complexification of LDTs, but also to the fact that LDTs are being used to detect high‐risk conditions.

### 4.5 General versus patient‐specific outputs

Regulators have expressed their intention to exercise oversight over apps that provide user‐specific health information (e.g., risk of disease) and recommendations (Gutierrez, 2010; U.S. Food and Drug Administration, 2015). Generally, then, an app that is intended to make “general recommendations,” or which addresses a “group” of persons is less likely to be a diagnostic device.

Developers of genomic communication and interpretation software may in some cases be able to avail themselves of these exceptions, but they face significant uncertainty, especially as both genomics and software tools become more complex, and where software is distributed globally.

## 5 RISK CLASSIFICATION AND REGULATORY REQUIREMENTS

The rules or controls that apply to an MD/IVD in a particular jurisdiction depend on the device's risk classification (Table 2). The risk class is important to know in advance of software development and distribution. Regulatory requirements include general controls, labeling, premarket controls (notification or approval), general safety and performance requirements, and postmarket surveillance. In the EU, MDs that conform to regulatory requirements are labeled with the CE Marking (“Confirmité Européene”). General controls are the least stringent regulatory layer, and will generally be the sole applicable regulation when a MD is of the lowest risk class (IMDRF SaMD Working Group, 2013). Yet even general controls can be quite onerous. Typically, they pertain to labeling; adulteration; misbranding; establishment registration; device listing; premarket notification; banned devices; notification and repair, replacement, and refund; records and reports; restricted devices; and good manufacturing practices. Beyond general controls, the regulator may exercise specific controls, such as premarket approval and postmarket surveillance over medium‐ to high‐risk devices. According to the FDA, premarket approval “is based on scientific evidence providing a reasonable assurance that the device is safe and effective for its intended use or uses” (U.S. Code of Federal Regulations, 2017). In Europe, premarket assessment of conformity is generally delegated to Notified Bodies (EU MDR, EU IVDR, Annex VII). From a practical perspective, it is key for a genomic software developer to determine if the software is a MD or an IVD, and how the software might be categorized with regards to risk, across jurisdictions where the software is made available.

**Table 2:** Risk Classification for Medical Devices and IVDs

**FDA**	Class I (low) Most are exempt from 510(k) Premarket Notification Bandages	Class II (medium) Most require Premarket Notification 510(k) Surgical mask		Class III (high) Pre‐market approval required Implantable neuromuscular stimulator; NGS‐based test for germline disease
**EU Medical Device Regulation**	Class I (low) Self assessment	Class IIa (medium) Surgical clamps	Class IIb (medium‐high) Condoms	Class III (high)
		“notified body” approval required		
**EU In Vitro Diagnostic Device Regulation**	Class A (low) Self assessment Specified products, e.g., for general laboratory use (Rule 5)	Class B (medium) Default category (Rule 6)	Class C (medium‐high risk to individual/public health) Human genetic testing (Rule 3)	Class D (high risk to individual/public health) E.g., Detection of a transmissible agents for transfusion or potential for life‐threatening disease (Rule 1) Specific blood grouping tests (e.g., ABO) (Rule 2)
		“notified body” approval required		
**Health Canada**	Class I No medical device licence required Laboratory equipment and general diagnostic reagents	Class II IVDs that detect infectious agents that are not easily propagated in a population	Class III IVDs where erroneous result would put patient in a life‐threatening situation	Class IV IVDs used for diagnosis of life‐threatening diseases
		‐ Medical device licence required ‐ IVDs must comply with quality management system requirements ‐ Must also comply with additional quality system requirements of the Canadian Medical Devices Regulations		

Regulatory frameworks typically default to a risk‐based approach to regulation. Stand‐alone software, if qualifying as a MD/IVD, tends to fall in lower risk classes (unless errors could lead to immediate danger) (Quinn, 2017). Generally, whether the device is intended for use in a clinical context or for use by the public (e.g., self‐testing) can affect risk categorization if risks are higher when inexperienced lay people use the app (IMDRF SaMD Working Group, 2014). The IMDRF risk‐based approach has defined two general criteria for determining the risk class of software (IMDRF SaMD Working Group, 2014). The first factor is the significance of the information provided by the software to the healthcare decision. Software poses a high risk if outputs are used in the contexts of treatment or diagnosis to inform an immediate or near term action. Where outputs drive clinical management, by simply aiding in treatment or diagnosis, software is considered medium risk. Low risk software does not trigger an immediate action, and merely informs treatment or diagnostic options, or helps to aggregate relevant information. The second factor is the state of the healthcare situation or condition. In critical contexts, timely action is vital to avoid death, disability, or serious health deterioration. In serious contexts, timely intervention is important to mitigate long‐term irreversible health consequences. In nonserious contexts, timely diagnosis or treatment is important but not critical to mitigate long‐term irreversible consequences. More detail on these factors is available in Box 1. Examples of software classified according to risk are available in Box 2.

BOX 1 **Examples of medical devices and risk classifications**

The following examples of medical device and risk classification were drawn from the IMDRF international guidelines based on their relevance to genetic interpretation (IMDRF Software as a Medical Device (SaMD) Working Group, 2014). They are organized in general risk classes. Roughly concordant risk classes in specific jurisdictions are available in Table 2. These examples are based on a generic risk‐based approach, and predate the new European regulations. Following the IVDR, compulsory from 2022, readers should note that genetic IVDs in Europe will be automatically considered as Class C (medium–high risk) devices.

**Not a Medical Device**

Software that provides parameters that become the input for software is not software if it does not have a medical purpose. For example, a database including search and query functions by itself or when used by software is not software.

**Class I**

Software that uses nongenetic data from individuals for predicting risk score (functionality) in healthy populations for developing the risk (medical purpose) of migraine (nonserious condition).

**Class II**

Software that uses nongenetic data from individuals for predicting risk score for developing stroke or heart disease for creating prevention or interventional strategies.

**Class III**

Software that is intended as a radiation treatment planning aid by using information from a patient and provides specific parameters that are tailored for a particular tumor and patient for treatment using a radiation medical device.

Software that uses data from individuals for predicting risk score in high‐risk population for developing preventive intervention strategies for colorectal cancer.

BOX 2 **IMDRF Stand‐alone software risk class subfactors**

If stand‐alone software qualifies as a medical device, the following factors may be influential in determining potential impacts on patient safety:

- The type of disease or condition
- Fragility of the patient with respect to the disease or condition
- Progression of the disease or the stage of the disease or condition
- Usability of the application
- Designed toward a specific user type
- Level of dependence or reliance by the user upon the output information
- Ability of the user to detect erroneous output information
- Transparency of the inputs, outputs, and methods to the user
- Level of clinical evidence available and the confidence on the evidence
- The type of output information and the level of influence on the clinical intervention
- Complexity of the clinical model used to derive the output information
- Known specificity of the output information
- Maturity of clinical basis of the software and confidence in the output
- Benefit of the output information versus baseline
- Technological characteristics of the platform, the software are intended to operate on
- Method of distribution of the software.

Other aspects that are not included in the two major factors (e.g., transparency of the inputs used, technological characteristics used by particular software, etc.), although still important, do not influence the determination of the category of software. These other aspects influence the identification of considerations that are unique to a specific approach/method used by the manufacturer of a particular category of software. For example, the type of a platform used in the implementation of software may create unique considerations. These considerations can also vary with the capabilities of the manufacturer or by the rigor of the process used to implement the software. Appropriate considerations of these aspects by the manufacturers, users, and other stakeholders can significantly minimize patient safety risks (IMDRF SaMD Working Group, 2014).

Some significant distinctions to the default risk‐based approach must be noted. First, the FDA's future approach to patient decision supports software may be at odds with the IMDRF's risk‐based framework. Specifically, while interpreting the statutory exemptions of clinical decision support software created by the Cures Act, the FDA has implemented a nonstatutory, policy exemption for patient decision support software (U.S. Food and Drug Administration, 2017c). While this policy exemption mirrors the statutory exemption for clinical decision support software, it is not explicitly risk‐based. Instead, the Clinical Decision Support Guidance suggests that software will be regulated if it fails to provide a sufficient basis for its recommendation, regardless of risk. Predictably, stakeholders were concerned. The American Medical Informatics Association, for instance, highlighted the lack of discussion on “variability across patient populations in their health literacy” (American Medical Informatics Association, 2018). Risk assessment may also be affected by statutory or discretionary genetic exceptionalism, reflecting a belief that genetic data is more special (i.e., risky) than other health data. The EU IVDR, for example, adopts a deterministic approach to classify the risk of genetic IVDs, assigning all genetic tests to the same medium–high risk class regardless of whether the test is predictive or diagnostic (EU IVDR, Annex VIII). This classification mandates “comprehensive requirements for clinical evidence of performance and effectiveness” before a genetic IVD can receive a CE Marking and be placed on the market (Hall et al., 2018). This may result in procrustean regulation of genomic software: strict rules if they qualify as IVDs or no rules at all. In Canada, risk classification of MD/IVDs is rule based, and focused primarily on the device's intended use. While Health Canada is currently preparing draft guidance specifically for software as a MD (Health Canada, 2018), the current practice seems clear: the importance of the information transmitted by the device is secondary to the device's intended use (Health Canada, 2015, 2016). If nothing else, this increasing regulatory differentiation across the various jurisdictions may perhaps signal the need for further IMDRF harmonization.

## 6 RISK MANAGEMENT STRATEGIES

Genomic software developers have ethical and regulatory duties to mitigate the risk that their software outputs prompt a harmful healthcare decision. Among these duties is to ensure that the appropriate uses and limitations of software applications are clearly and transparently described. This follows the general expectation for variant databases that they curate and share genomic data in a manner that is “truthful and not misleading … in language that is clear and understandable” (U.S. Food and Drug Administration, 2018d). General software design, development, and documentation practices should be followed, informed by the software's purposes and reasonable foreseeable uses (IMDRF SaMD Working Group, 2014). It may also be important to consider how changes to the software will be implemented, which depends on one's ability to control versions and successive implementations of the software. As software development is often an iterative process, adequate records of product performance, validation, and quality assurance should be kept. The general regulatory concept of postmarket surveillance applies helpfully to software. One example of a postmarket surveillance system is the UK Medicines and Healthcare products Regulatory Agency's Yellow Card scheme, used to monitor adverse drug reactions, MD adverse incidents, and counterfeit MDs. Similarly, developers should take steps to capture user feedback, which can help to better understand the human factors that may affect how software is ultimately used. “After all, even as computers become increasingly sophisticated, there will always be challenges at the human–computer interface, where unintended “friction” between human and machine can rapidly defeat the potential benefits” (Senger & O'Leary, 2018). An intuitive user design, across platforms on which the software is likely to be implemented, can also ensure the software is appropriately used. Building on the concept of proper “labeling,” careful consideration should be given to the software's terms of use. These terms could include clear information about any quality limitations of algorithms or the underlying genomic data, which can help “users question the validity of output of the [s]oftware and avoid making incorrect or poor decisions” (IMDRF SaMD Working Group, 2014). Security to ensure the availability, integrity, and confidentiality of data is also an important consideration, especially where software ingests user data as inputs. Data protection laws and related duties of confidentiality for personal data may also apply.

Encouragingly, regardless of regulatory application, there exist strong incentives for developers to establish risk management practices. Among other incentives, responsible development and dissemination of software may decrease the chance that the software attracts the attention of regulators or lawmakers, or lessen the burden of transitioning to regulated status. In fact, the prospects of streamlined regulation for responsible developers may no longer be a mere mirage. The FDA has begun work on the Digital Health Software Precertification (Pre‐Cert) Pilot Program. This Pre‐Cert Program is intended to provide more “streamlined and efficient regulatory oversight of software‐based devices developed by manufacturers who have demonstrated a robust culture of quality and organizational excellence, and who are committed to monitor real‐world performance of their products once they reach the US market” (U.S. Food and Drug Administration, 2018a). Critically, under this program, the focus of the FDA is primarily the software developer, not the product. Indeed, upon completion of the Pre‐Cert pilot program, Europe may soon follow with its own precertification analog. Assuredly, the promise of a more streamlined regulatory process could motivate software developers to foster an internal culture of responsible and responsive development.

## 7 CONCLUSIONS

It is not straightforward to determine if MD/IVD regulations are applied or should be applied to genomic software tools. The rules are in flux as law makers and regulators struggle to keep up with the “appification” of health and medicine, and to promote innovative device development while also protecting patients from harm. Appification has spurred incredible innovation, in part by democratizing both device “manufacture” —beyond well‐resourced companies to include small scale developers—as well as device selection and use beyond medical institutions and professionals to include patients and lay users (Quinn, 2017). It has also fundamentally changed the nature and complexity of risks of devices, raising daunting conceptual and practical challenges for regulators. The data deluge of genomics is ripe for software intermediation, but genomic complexity and unfamiliarity may mean such tools are subject to exceptional regulatory scrutiny.

At the heart of the software as MD question lies the policy tension between access to innovative tools to interact with genetic information on one hand, and overexposure of the public to risk on the other. This is part of a broader debate about access to and the quality of our accumulating genomic knowledge. Previously, we have argued that public variant databases remain unlikely to attract legal duties toward patients for data quality and timeliness, despite their increasing medical importance. These duties continue to rest primarily with physicians, and in some cases laboratories (Thorogood, Cook‐Deegan, & Knoppers, 2017). Sharing genomic software in addition to data may change this equation by establishing novel legal relationships between databases and users. Regardless, many variant databases are keen to adhere to curation and management standards, such as those recently released by the FDA (U.S. Food and Drug Administration, 2018d). Compliant databases can receive FDA recognition, meaning their data can be used as evidence in sequencing test regulatory submissions. While this paper has focused on MD/IVD regulation of software, broader legal questions—such as who bears responsibility for the quality and currency of genomic data used in health care (Stevens, Senner, & Marchant, 2017)—merit further exploration.

## CONFLICT OF INTERESTS

The authors have no known conflicts of interest.
